# Regulatory Network of Serine/Arginine-Rich (SR) Proteins: The Molecular Mechanism and Physiological Function in Plants

**DOI:** 10.3390/ijms231710147

**Published:** 2022-09-05

**Authors:** Xiaoli Jin

**Affiliations:** Departmeng of Agronomy, College of Agriculture and Biotechnology, Zhejiang Key Laboratory of Crop Germplasm, Zhejiang University, Hangzhou 310058, China; jinxl@zju.edu.cn

**Keywords:** SR proteins, alternative splicing (AS), pre-mRNA splicing, post-splicing, translation regulation

## Abstract

Serine/arginine-rich (SR) proteins are a type of splicing factor. They play significant roles in constitutive and alternative pre-mRNA splicing, and are involved in post-splicing activities, such as mRNA nuclear export, nonsense-mediated mRNA decay, mRNA translation, and miRNA biogenesis. In plants, SR proteins function under a complex regulatory network by protein–protein and RNA–protein interactions between SR proteins, other splicing factors, other proteins, or even RNAs. The regulatory networks of SR proteins are complex—they are regulated by the SR proteins themselves, they are phosphorylated and dephosphorylated through interactions with kinase, and they participate in signal transduction pathways, whereby signaling cascades can link the splicing machinery to the exterior environment. In a complex network, SR proteins are involved in plant growth and development, signal transduction, responses to abiotic and biotic stresses, and metabolism. Here, I review the current status of research on plant SR proteins, construct a model of SR proteins function, and ask many questions about SR proteins in plants.

## 1. Introduction

**Figure 1:**
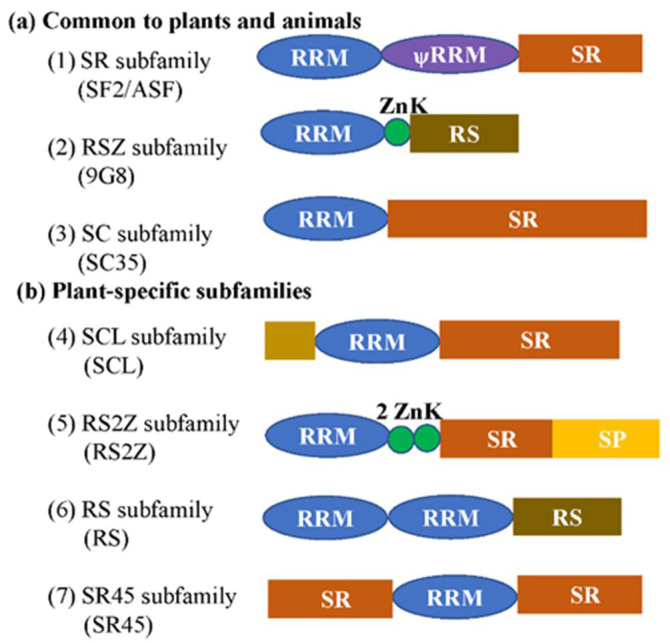
Schematic representation of plant SR proteins. (**a**) Three subfamilies are common to plants and animals. (**b**) Subfamilies that are specific for the plant kingdom. The subfamily names without brackets are from [5], while ones with brackets are from [6]. RRM, RNA recognition motif; ѱRRM, RRM (contains the SWQDLKD motif, which is present in all SF2/ASF homologs); SR, domain rich in serine-arginine dipeptides; RS, domain rich in arginine and serine; ZnK, zinc knuckle of CCHC type; SP, domain rich in serine and proline. The brown rectangle indicates the rare domains which could be diverse.

In eukaryotes, genes undergo pre-mRNA splicing, post-splicing, and translation from DNA to mature protein. A family of proteins called serine/arginine-rich (SR) proteins are the most extensively studied in animals and to a lesser extent in plants. They play an important role as non-snRNP proteins [1]. The SR protein family comprises a number of phylogenetically conserved and structurally related proteins with a characteristic domain rich in arginine and serine residues, known as the RS domain. Manley and Krainer defined an SR protein as any protein with the following minimal attributes: one or two N-terminal RBDs (PF00076), followed by a downstream RS domain of at least 50 amino acids with >40% RS content, characterized by consecutive RS or SR repeats [2]. In metazoans, the new nomenclature is based on the root “SRSF” (SR splicing factor) followed by numbers. In the plant SR protein community, a standard nomenclature is used (Figure 1). The family of serine/arginine-rich proteins (SR proteins), as a type of splicing factor, has recently attracted attention in plants. The SR proteins form a complex network by interacting with small nuclear ribonucleoprotein particles (snRNPs), SR proteins, kinases, the cap-binding complex (CBC), C3H-type zinc finger protein, helicases, cyclins, and cyclophilins, etc. [3]. These regulation target genes of SR proteins are orchestrated through an extensive protein–RNA interaction network involving *cis*-elements within the pre-mRNA and *trans*-acting factors that bind to these *cis*-elements [4]. The aim of this article is to review the regulatory network of SR proteins and the molecular and physiological mechanism based on updated publications. In addition, I will discuss how the regulation network of SR proteins leads to plant growth and development, signal transduction, and responses to abiotic and biotic stresses.

## 2. The SR Protein Family and Its Structure

The family of SR proteins (SRSF proteins) has been widely and deeply studied in mammalian pre-mRNA splicing. Recently, a relevant study in plants has drawn attention, as SR proteins have been linked to important roles in gene regulation during development and in response to environmental stress [7]. Until now, 19, 24, 21, 18, and 24 SR proteins have been identified in Arabidopsis, rice, maize, sorghum, and *Populus trichocarpa*, respectively [8,9,10]. Through genome-wide analysis, more SR proteins were identified in longan [11], maca [12], and other plants [10]. Details of the SR members in plants are presented in Table S1.

SR proteins belong to seven plant subfamilies, which contain one or two RNA recognition motifs (RRMs) in the N-terminus and one arginine/serine-rich (RS) domain in the C-terminus. In addition, some SR proteins have a specific structure, Zn-knuckles, RGG box (a prion-like domain, and a nuclear shuttling sequence), or two RS domains (Figure 1). The RRM domains provided RNA-binding specificity and the RS domain functions as a protein-interaction domain. In Arabidopsis, the RRM1 motif of plant-specific RSp29 is essential for the increased efficiency of splicing, whereas RRM2 is indispensable for the enhancement of splicing by RSp29, but is not involved in splice site selection [13]. The RRM1 or RRM2 of SR34 are indispensable in exclusive protein nuclear localization and speckle-like distribution. The RS domain is involved in protein–protein and protein–RNA interactions, and the ESE-bound RS domain functions by contacting the branch-point to promote pre-spliceosome assembly [14]. Until now, the role of the Zn-knuckle has less well understood, and the functional relationship between RRM and Zn-knuckle domains has still to be explored.

## 3. SR Proteins and Their Network of Interactions

**Figure 2:**
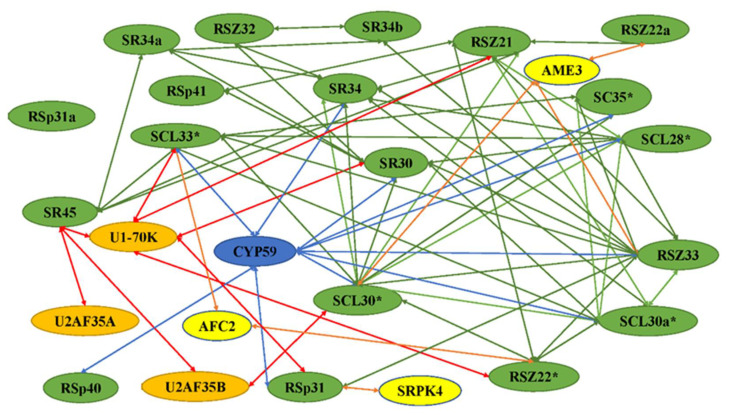
Network of interactions among SR proteins and other spliceosomal proteins. These interactions were identified using yeast two-hybrid analysis and/or in vitro protein–protein interaction assays. The interaction of SR protein with itself is indicated by asterisks. The green, red, orange, and blues arrows indicate the SR proteins interacting with SR proteins, splicing factors, protein kinases, and cyclophilin-like proteins, respectively. All SR proteins are shown in green. The snRNP proteins U1 and U11, and splicing factor U2af small subunits U2AF35A and U2AF35B are indicated in orange. Protein kinases and cyclophilin-like proteins are shown in blue and yellow, respectively.

When the SR proteins function, they form complexes with other proteins. The protein–protein interaction in plant SR proteins have not only revealed interactions between SR proteins and other splicing factors, but also established the regulation mechanism of SR proteins (Figure 2). Given the current scenario, the different interaction proteins are illustrated, and partial interaction proteins which have been identified to play a role in plants are summarized in the following sections.

**Figure 3:**
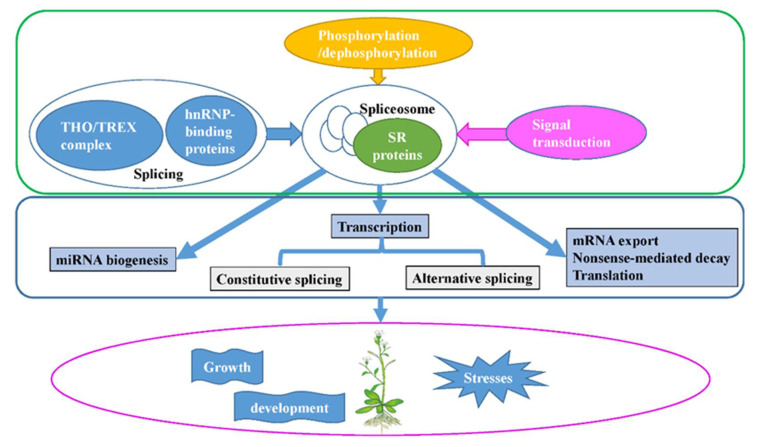
Schematic of the functional processes of SR proteins. SR proteins, which form the spliceosome by interacting with snRNPs, themselves, or other SR proteins, play significant roles in constitutive and alternative pre-mRNA splicing, and are involved in post-splicing activities, such as mRNA nuclear export, nonsense-mediated mRNA decay, mRNA translation, and miRNA biogenesis. Meanwhile, SR proteins are regulated by splicing, phosphorylation/dephosphorylation, and signal transduction. By their molecular function and regulated network, SR proteins take part in plant growth and development, and responses to abiotic and biotic stresses.

The SR proteins are involved in the spliceosome by interacting with snRNPsas splicing factors, and many interactions with themselves or other SR proteins have been revealed (Figure 2). The splicing of nuclear pre-mRNAs takes place in a multi-component complex called the spliceosome (Figure 3). Normally, the proteins form a major spliceosome, which consists of a pre-mRNA, five snRNPs, and other non-snRNP splicing factors including SR proteins. The atSR45a-1a and atSR45a-2 proteins interact with U1-70K and U2AF^35^b during spliceosome assembly. The two isoforms also interact with themselves, other SR proteins, and PRP38-like protein [15]. Like the major spliceosome, most SR proteins have been found to interact with U11-35K, form minor spliceosomes, and play a role in splicing of minor introns [16]. The co-localization in nuclear speckles, and the interaction and co-expression between U2AF65a and SC35/SCL proteins have been observed, indicating the relationships between snRNP and SR proteins for the spliceosome [17].

The SR proteins have been found to interact with themselves or other SR proteins. atRSZ33 interacted with atSRp34/SR1, atRSZp21, atRSZp22, atSCL28, atSCL30, and atSCL33/SR33. SCL30a and atSC35, belonging to the SCL sub-family, were also found to interact with atRSZ33 [18]. TaRSZ38 was found to not only interact with snRNPs but also with SR proteins and other proteins [19]. SR34 could interact with SR45 [20]. Interestingly, the members of different SR protein sub-families localize into distinct populations of nuclear speckles with no, partial, or complete co-localization, indicating that SR proteins are partitioned into distinct populations of nuclear speckles to allow a more specific recruitment to the transcription/pre-mRNA processing sites of particular genes depending on cell type and developmental stage [21].

In addition to the action of more general splicing factors, SR proteins have been found to bind to more specific proteins, which are often regulated in a cell-, tissue-, or developmental-stage-specific, or stress manner. These proteins included novel proteins potentially involved in splicing regulation, kinases, CBC, C3H-type zinc finger protein, helicases, cyclins, and cyclophilins (Figure 2). The interactions of SR proteins with other proteins indicates that SR proteins play multiple functions or are regulated by different pathways. In plants, the interaction of SC35 and SCL proteins with NRPB4 has been observed. Flag-fused SCL28 and SCL30 were observed to co-immunoprecipate with YFP-fused NRPB4, but not with SCL30a, SCL33, or SC35, possibly due to weak interactions between themselves and NRPB4, suggesting they may have a function in the coordination of transcription and splicing [17]. Some studies have reported that kinases are involved in the interaction with SR proteins. For example, SRPK4, a member of a family of SRPK kinases in plants, interacted with RSp31 and other SR proteins, suggesting that SRPK4 is one of the kinases regulating SR protein function [3,22]. RSZ33 interacted with cyclin-dependent kinase G1 (CDKG1), through forming the nuclear speckles and residing in the same complex. CypRS64 interacts with a subset of Arabidopsis SR proteins, including SRp30 and SRp34/SR1. In addition, both cyclophilins interact with U1–70K and U11–35K, which in turn are binding partners of SRp34/SR1. AtCyp59 was identified as an interacting partner of the Arabidopsis SR protein SCL33/SR33 [16]. AtCyp59 dots have been found to localize on the periphery of speckles in which SR proteins are found. SR45a directly interacted with the CBC subunit cap-binding protein 20 (CBP20), which was originally identified through its role in pre-mRNA splicing and m7G-cap structure binding [23]. SR1, and other proteins including SRm102 and U1-70K, belonging to the components of the U1 spliceosome interacted with GDS1, which encodes a C3H42 protein, in the nuclear speckle compartments, suggesting that SR1 might play a significant role in splicing by forming spliceosome with other proteins [24]. The splicing factor SR34 colocalizes with SmD1, a component of the Smith (Sm) complex, in posttranscriptional gene silencing (PTGS) in nucleoplasmic speckles [25]. OsFKBP20-1b, belonging to the immunophilin family, interacted with OsSR45 in both nuclear speckles and cytoplasmic foci, and played an essential role in post-transcriptional regulation of abiotic stress response [26]. The SR protein interacted with highly ABA-induced 1 (HAI1) protein in nuclear speckle during drought stress [27]. The interaction network is presented in Table S2 [15,16,21,22,28,29,30,31,32,33,34,35,36,37,38,39,40,41,42,43,44,45,46,47,48,49,50]. 

## 4. SR Proteins and Their Molecular Function

The SR proteins participate in constitutive and alternative pre-mRNA splicing, and post-splicing activities: mRNA nuclear export, nonsense-mediated mRNA decay (NMD), mRNA translation, and miRNA biogenesis, etc. A number of studies focus on the Arabidopsis SR proteins (Table S3). The details are listed below

### 4.1. SR Proteins and Transcription

Pre-mRNA splicing, which is a posttranscriptional issue regulated by the spliceosome, is an essential step in the flow of genetic information in virtually all eukaryotes. Pre-mRNA splicing not only modulates the constitutive gene expression, but also generates multiple transcripts from the same genome sequence. Thus, pre-mRNA splicing is an important modulator of gene expression that can increase proteome diversity and regulate mRNA levels [6,13]. The molecular mechanism of SR protein involved in the pre-mRNA splicing was summarized by Reddy and Shad Ali, [6] (Figure S1). The SR proteins bind to sequences in exons, and then recruit and stabilize U1 snRNP on the 5′-splice site and the heterodimeric U2AF complex to the 3′-splice site and U2 snRNP to the adjacent branch point. As mentioned above, the SR proteins contribute to the splicing by forming the spliceosome to a spectrum of target genes. Using RNA immunoprecipitation followed by high-throughput sequencing, over 4000 RNAs were identified to be directly or indirectly associated with SR45 in *Arabidopsis thaliana* [51]. SR45-1 was also found to broadly target alternative splicing (AS) in vivo, including that of the SR45 pre-mRNA itself [52]. In constitutive splicing, the SR proteins function on the RNA in a non-specific manner. SR45.1 promoted the constitutive splicing of SR30 mRNA [28]. AS relies upon the interaction of SR proteins with RNA regulatory sequences (exonic splicing enhancers, intronic splicing enhancers, exonic splicing silencers, and intronic splicing silencers). For example, the SC35 and SCL proteins interact with subunits of U1 and U2 snRNPs by forming a spliceosome. A short AGAAGA motif on a subset of target genes was identified to be a specific SC35/SCL protein-binding sequence by the spliceosome [17]. OsRSp29 and RSZp23, enhanced the splicing efficiency and changed the alternative 5′ splice sites of Wx^b^ intron 1 [13]. SR45 recruits U1snRNP and U2AF to 5′ and 3′ splice sites, respectively, by interacting with pre-mRNA, U1-70K, and U2AF35 and modulates AS [47].

### 4.2. Role of SR Proteins in mRNA Export, NMD, and Translation

In addition to constitutive pre-mRNA splicing and alternative splicing, the SR proteins also participate in mRNA nuclear export, NMD, and translation, which occurs after splicing. Recently, the SR proteins have been found to function as a connector from the nucleus to the cytoplasm. Most SR proteins dynamically localize to speckles and chromatin in the nucleus, whereas some SR proteins can export out to the cytoplasm and shuttle continuously between the nucleus and the cytoplasm [11]. In Arabidopsis, the SR proteins localize into nuclear irregular dynamic domains similar to speckles, with no, only partial, or complete co-localization [6]. The localization and shuttling behavior of SR proteins indicates that they have a functional role between the nucleus and the cytoplasm. Some SR proteins function as the receptors for mRNA nuclear export by interacting with the export receptor nuclear export factors [53,54]. The SR protein SR33 co-localized with HPR1 in nuclear speckles played a role for HPR1 in RTE1 expression during transcription elongation and less likely during export [55]. Localization studies revealed that Arabidopsis SmD1b colocalized with the splicing-related factor SR34 in nuclear speckles. The results suggested that SR34 could be involved in the same function with SmD1, which interplays with splicing, RNA quality control (RQC), and posttranscriptional gene silencing (PTGS) [25].

The SR proteins were also detected in NMD, a special type of AS event occurring at the 3′ splice site, and in translation, and were correctly processed to prevent the production of truncated proteins. In Arabidopsis, 13 SR genes are alternatively spliced to generate 75 transcripts, of which, 53 contain a premature termination codon (PTC) and about half of the PTC-containing splice forms were confirmed to be the targets for degradation through NMD [56]. In the maca transcriptome, SR proteins were identified as another important component in NMD [12].

### 4.3. SR Proteins Participate in miRNA Biogenesis

MiRNA biogenesis is highly regulated at the post-transcriptional level. In metazoans, the SR (SRSF) protein family member promotes pre-miRNA processing of miRNA biogenesis [57,58,59]. A genetic variant in a conserved region within the terminal loop of miRNA, which causes a reorganization of the RNA secondary structure and promotes the interaction with SRSF3, an SR protein, leading to the increased levels of miRNA [57]. Ratnadiwakara et al., summarized that SR proteins have recently been implicated in miRNA biogenesis [59]. Normally, SR proteins bind to the flanking ssRNA ∼18 bp downstream of the stem loop of the pre-miRNA, which could be a specific sequence motif recognized by the SR proteins, and enhance pre-miRNA cleavage, and form matured miRNA (Figure S2). However, to date, little attention has been paid to the mechanism. Thus, the mechanism of miRNA biogenesis regulated by SR proteins in plants is still unknown. HOS5, RS40, and RS41, previously shown to be involved in pre-mRNA splicing, affect the biogenesis of a subset of miRNA. Differently with the SR proteins involved in pre-miRNAs, the SR proteins bind to both intron-less and intron-containing pre-miRNAs during microRNA splicing [60].

## 5. The Regulation of SR Proteins

SR proteins, as splicing factors, participate in other types of gene regulation, including transcription and post-transcription. Until now, little work has been carried out on the regulation on the SR proteins themselves. Herein, I summarize the regulatory mechanisms of SR proteins: constitutive pre-mRNA splicing and AS, mRNA nuclear export, NMD and translation through the THO/TREX complex and hnRNP-binding proteins (RBPs), dynamic phosphorylation and dephosphorylation, and signal transduction pathways.

### 5.1. The Splicing of SR Proteins

(1) The splicing of SR proteins is regulated by the THO/TREX complex

The SR proteins participate in constitutive pre-mRNA splicing and AS, mRNA nuclear export, NMD, and translation not only of other genes but also of themselves. The components of the THO/TREX complex have specific roles in the transcription or export of selected genes, and in translation, RNA decay, and small interfering RNA-dependent processes in plants [54,55]. The SR proteins are present in similar location to the components of the THO/TREX complex [55]. EMU, an Arabidopsis homolog of the yeast THO complex member HPR1, is involved at least in the regulation of alternative pre-mRNA splicing of SR proteins [61]. 

(2) The splicing of SR proteins is regulated by RBPs

The SR proteins act as splicing repressors and are required for the regulation of splicing by binding to their transcripts, generating NMD splice variants, maintaining homeostatic protein expression, and downregulating proteins [1]. The activity or the isoforms of some SR proteins can be regulated by members of the hnRNPs A/B family and by other SR proteins, or even by themselves. It has also been shown that manipulating the expression of SR proteins alters the splicing of their own pre-mRNA and other SR genes [62]. The regulation of SR proteins is concentration-dependent. In Arabidopsis, overexpression of atSRp30 resulted in AS of several SR proteins, including *atSRp30* itself and *atSRp34*/*SR1* [63], whereas the overexpression of atRSZ33 caused changes in the splicing pattern of its own and other SR genes including *atSRp30* and *atSRp34/SR1* [62]. SR45 can bind in the 5′ region of SR30 intron 10, which recruits U1snRNP and U2AF to 5′ and 3′ splice sites, respectively, by interacting with pre-mRNA, U1-70K, and U2AF35 and modulates AS of SR30 [47].

### 5.2. Phosphorylation and Dephosphorylation 

The dynamic phosphorylation and dephosphorylation of SR proteins plays a role in their subcellular distribution and protein–protein interactions, and in their post-splicing activities in mRNA export, stability, and translation. During rhizobia colonization and infection of *Lotus japonicas*, most SR proteins were found to be multiply phosphorylated [64]. The SRPK4 (SR protein-specific kinase 4) and MAPKs (mitogen-activated PKs) were found to phosphorylate SCL30, but SCL30 was targeted by different PKs [3]. Intriguingly, ATP depletion could indeed alter the phosphorylation levels of proteins and change protein interactions, which could in turn modulate and modify the dynamic properties of SR proteins. A dynamic cycle of phosphorylation and dephosphorylation is required for pre-mRNA splicing, being related, at least in part, to the phosphorylation status of SR proteins. The transcription activity of the cell and protein (de)phosphorylation regulates the intranuclear distribution of SR 45 [65]. The RNA splicing and brassinosteroid (BR) signaling pathways were extensively affected by phosphorylation, and most SR proteins were multiply phosphorylated [64]. 

### 5.3. Signal Transduction Pathways

Importantly, SR proteins are extensively regulated by signal transduction pathways, whereby signaling cascades, temperature, phyto-hormones, and light, etc., can link the splicing machinery to the exterior environment. Temperature stress dramatically altered the splicing of pre-mRNAs of several SR genes, whereas hormones altered the splicing of only three SR genes [66]. The SR proteins could act as central coordinators of plant abiotic stress responses by targeting key components of phyto-hormone signal transduction. The mutant of *RS40* and *RS41*, is hypersensitive to abscisic acid (ABA) and salt stress [67]. *SR34*, *SR34b*, *SCL30a*, *SCL28*, *SCL33*, *RS40*, *SR45,* and *SR45a* are regulated by ABA which could be involved in ABA-mediated stress [68,69]. Meanwhile, ethylene regulates the SR proteins by phosphorylation. Tobacco PK12 was found to be co-localized with arSRp34/SR1, and phosphorylated SR proteins [70]. SR33 co-localized with HPR1 in nuclear speckles, which was required for RTE1 over-expresser (RTE1ox) ethylene insensitivity at the seedling but not adult stage [55]. Light can affect SR proteins, both as an environmental signal and as an energy source. Intense light-stress changed the splicing pattern of SR30, atSR45a, atSR31, and atU2AF65A, while the levels of the transcripts atSR45a, atSR30, and SF2/ASF-like SR were increased by high-light irradiation and salinity stress [71]. The spectrum also affect the AS change of SR proteins. For example, red light mediated AS changes of AtSR30, AtSR31, AtSR31a, and AtU2AF65A [72].

## 6. SR Proteins Function in the Growth and Stress Response

The SR proteins in plant have been found to participate in root formation, seed dormancy, seedling development, flowering, pollen germination, pollen wall formation, etc. [63,73]. *atSRp30* expresses alternatively spliced mRNA isoforms that are expressed differentially in various organs and during the developmental process [63,74,75,76]. Normally, the SR proteins function in the growth or stress response by affecting pre-mRNA splicing, post-splicing, translation, and regulation of the target genes. Numerous SR proteins cooperated with DHT1 to regulate posttranscriptional splicing and SL signaling, which resulted in a change in tilling number and height in rice [74]. In *Popolus trichocarpa*, most PtSR genes (~83%) responded to at least one stress (cold, drought, salt, SA, MeJA, or ABA) [9]. The *atSR45* knock-out mutant, displayed late flowering by influencing the autonomous flowering pathway, and altered leaf and flower morphology by changing splicing-regulated splicing patterns of flowering regulators (*FCA*, *MAF2,* and *FLM*) and the expression profiles of several SR genes (*atSRp30*, *atRSZp22a*, and *atSCL33/SR33*) [75,76]. The SR45 protein negatively regulated early seedling development by affecting glucose and ABA signaling [69]. The different isoforms of SR 45 showed different functions: SR45.1 functioned in flower development and salt tolerance, while SR45.2 played a role in root growth [77,78]. Overexpression of atSRp30 resulted in morphological and developmental changes, displaying mostly late flowering [63]. atSRp30 affected the AS patterns of several genes, *atRSp31*, *atU1-70K,* and *atSRp34/SR1*, and its own pre-mRNA. In particular, elevated levels of atSRp30 changed the splicing pattern in *atSRp34/SR1* in a way that mRNA1 encoding the full-length protein was decreased, but mRNA3 encoding a shorter protein was strongly increased. Moreover, the level of atSRp34 protein was downregulated, whereas these plants accumulated the shorter atSRp34 protein. *Arabidopsis thaliana* CDKG1 was recruited to U1 snRNP through RSZ33 to facilitate the splicing of CalS5, regulating the pollen wall formation [79]. LlSR28 altered F-actin dynamics probably through its AS activities to affect, directly or indirectly, the AS of AtVLN1 and the expression of different ABPs, which then affected the pollen germination [4]. Several SR proteins could affect growth as critical regulators of Zn, Mn, and P nutrition, and P uptake and remobilization between leaves and shoots in rice [80]. As mentioned above, the SR proteins interacted with kinases [3] and were regulated by phosphorylation and dephosphorylation [64]. Thus, the specific phosphorylation mechanism is involved in the SR protein, then differentially regulates the function of a plant splicing activator in physiologically and morphologically distinct plant tissues. Taken together, a tight control of SR protein levels plays important roles in particular cell or tissue types.

Besides the effect on the growth and development, the SR proteins work in response to stresses and hormones [81,82,83,84,85,86]. The hypersensitivity of expression regulation in response to environmental stresses of SR genes has been well studied in Arabidopsis and rice [66,81]. In Arabidopsis, 15 out of 19 SR (SR-like) genes were found to undergo AS regulation when the plant was subjected to abiotic stresses and phyto-hormone treatments. In rice, the variations were detected in the divergence in expression and splicing patterns of *SR* genes from seedlings of different rice ecotypes in response to hormones application and environmental stresses [81]. As the function of the isoforms of SR proteins is in growth and development, they contributed to stress response [78]. Some isoforms of SR proteins, including *SR32* and *RSZ23*, were differentially expressed under hypoxia treatment, suggesting that the change of AS in splicing components may be crucial in response to hypoxia stress during rice germination [87]. Overexpression of *MeRSZ21b* resulted in improved drought tolerance through modulating ABA-dependent signaling [88]. SR45a mediated salt-stress responses by directly interacting with the CBP20 [23], while RS40 and RS41 functioned as vital modulators of salt-stress responses [28,67]. The Cd-upregulated Arabidopsis SR34b gene is a regulator involved in splicing, mRNA stability, and protein accumulation of the IRT1 gene [89]. Moreover, the SR proteins including AtSR30, AtSR31, and AtSR31a, were mediated by red light [72]. Alterative splicing events of SR proteins occurred more frequently under high-temperature or cold treatment [90]. Transcript levels of several key genes involved in RNA processing were also affected by changes in storage temperature in tomato fruit [91]. More details about SR proteins participating in the heat shock were reviewed by Ling [7]. A group of SR genes underwent specific AS regulation by inducing (RS40, SR34a, RSZ22, and RSZ22a) or repressing (SR30, SR34, RSZ33, and SR45a) special RNA variants under heat stress [7]. 

Furthermore, some SR proteins were also involved in the biotic stress. AdRSZ21 from *Arachis diogoi*, resulted in HR-like cell death [92]. In *Brachypodium distachyon*, the temporal splicing patterns of Bd-SCL33 followed the infection of Brachypodium with six additional viruses in diverse genera [93]. OsFKBP20-1b directly maintained protein stability of OsSR45 through OsFKBP20-1b-mediated RNA processing and contributed to stress adaptation in rice [26]. In summary, the findings refine knowledge of the SR-protein-coding genes and provide novel insights for enhancing plant resistance to environmental stress.

## 7. The SR-Protein-Dependent RNAs in a Post-Genomic Era

The SR proteins play essential roles in every aspect of RNA metabolism: pre-mRNA splicing, and post-splicing activities: mRNA nuclear export, NMD, mRNA translation, and miRNA biogenesis, etc., in plants. However, the SR-target RNAs and their characteristics are still poorly understood, especially the global analysis of RNA targets of SR proteins in plants. In the post-genomic era, novel genomic approaches, which facilitate the study of SR proteins, have been developed. McHugh et al. described methods for comprehensive experimental identification of RNA–protein interactions [94], which could be utilized in the study of SR proteins and their target RNA. Of all novel genomic approaches, RNA-seq and RNA immunoprecipitation sequencing (RIP-seq) are the most possible approaches for determining the targets of the SR proteins. By RNA-seq, SC35 and SCL proteins were found to participate in the pre-mRNA splicing. In the *sc35-scl* mutant, 213 genes were found to show significant changes in AS, including alteration of all the common AS patterns and the expression levels of 1249 genes, indicating that OsSC35 was involved in constitutive and alternative pre-mRNA splicing. Motif analysis revealed that SC35 and SCL proteins recognize specific RNAs containing the AGAAGA motif [17]. In Arabidopsis, over 4000 RNAs that directly or indirectly associate with SR45 were identified using RNA immunoprecipitation (RIP) followed by high-throughput sequencing. Of them, 30% were abscisic acid (ABA) signaling genes. Most were derived from intron-containing genes, whereas 340 genes were derived from intronless genes. Moreover, four overrepresented RNA motifs that could recruit SR45 to the target RNAs were identified [51]. Similarly, by RNA-seq and RIP-seq, SR45 was found to recognize the GGNGG motif directly in inflorescence tissue [95]. While much effort has been put into developing methods to identify RNA and SR protein interactions, there are still significant challenges due to the limitations associated with widely used tools. For example, the SR proteins form spliceosomes to function on target RNAs, but there is no effective method to examine the protein complexes that interact with most RNAs. To date, the global target RNAs of SR proteins are little studied, and more novel genomic approaches should be applied in the protein–RNA interaction studies.

## 8. Concluding Remarks and Future Perspectives

SR proteins belong to a highly conserved family with RRM and RS domains across species. The SR proteins form complex networks to interact with other proteins and RNAs, regulating the constitutive pre-mRNA splicing and AS, mRNA nuclear export, NMD, and translation, thus affecting plant growth and development, signal transduction, and response to abiotic and biotic stress. The SR proteins can be regulated by splicing, phosphorylation and de-phosphorylation, and signal transduction pathways (Figure 3). In the future, the following issues should be addressed:

The SR proteins form spliceosomes to regulate the splicing. How do the spliceosomes and the splicing factors work in an orderly and subtle manner? 

The molecular mechanisms of SR proteins that are regulated by other proteins or signals should be investigated. Future identification of new splicing factors and their target mRNAs could improve our understanding of regulatory mechanisms of plant physiology, thus paving the way for new strategies to improve plant productivity in unfavorable environments.

The SR-target RNAs and their characteristics should be clarified to understand how the target RNAs regulate plant physiology. 

In short, SR proteins and their different isoforms play significant roles in plant growth and development, and in defense responses to various stresses. The molecular mechanisms of SR genes during plant growth, or in response to environmental stresses may be important for understanding the functions of SR genes, which may provide further functional elucidation of SR genes in plants. Moreover, plant SR proteins could function as central coordinators participating in diverse life processes of plants by affecting numerous target RNAs. Therefore, the SR proteins and their different isoforms have a pleiotropic effect on the plant physiology that would be helpful for plant breeders to optimize in their breeding schemes for specific cultivar improvement.

## Data Availability

Not applicable.

## Supplementary Materials

The following supporting information can be downloaded at: https://www.mdpi.com/article/10.3390/ijms231710147/s1.
