# Supplementary material for: Regulatory Network of Serine/Arginine-Rich (SR) Proteins: The Molecular Mechanism and Physiological Function in Plants
Source: Int J Mol Sci. 2022 Sep 5;23(17):10147. doi: 10.3390/ijms231710147 (PMC9456285; doi:10.3390/ijms231710147)
Supplement: Supplementary file 1 [file ijms-23-10147-s001.zip › Supplementary figures.pdf]

Supplementary figures

Figure S1

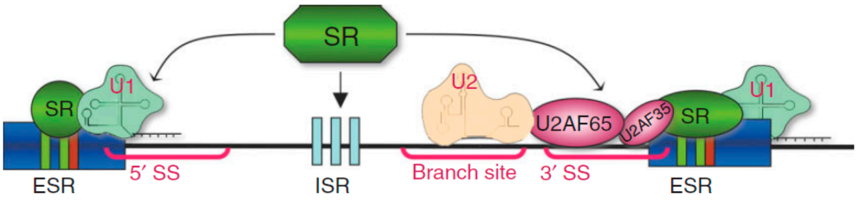

Reddy and Shad Ali, 2011

Figure S1 Roles of plant serine/arginine-rich (SR) proteins in precursor messenger RNAs (pre-mRNAs) splicing. SR proteins bind to sequences in exons, called exonic splicing regulators (ESRs), and then recruit and stabilize U1 small nuclear ribonucleoprotein (snRNP) on the 5' splice site (5' ss) and the heterodimeric U2AF complex to the 3' splice site (3' ss) and U2 snRNP to the adjacent branch point. They also mediate interaction between the U2AF complex and U1 snRNP across an exon or by binding to RNA sequences called intronic splicing regulators (ISRs) in introns to mediate interaction between the U2AF complex and U1 snRNP across introns. The figure is cited from Reddy and Shad Ali's paper[6].

Figure S2

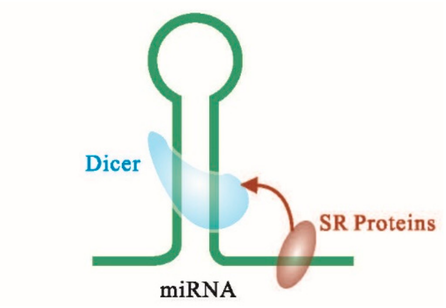

Figure S2 Post-transcriptional regulation of miRNA processing by SR proteins. SR proteins bind to the flanking sequence downstream of the stem loop of pre-miRNAs and enhance pre-miRNA cleavage.
