# Supplementary material for: Regulatory Network of Serine/Arginine-Rich (SR) Proteins: The Molecular Mechanism and Physiological Function in Plants
Source: Int J Mol Sci. 2022 Sep 5;23(17):10147. doi: 10.3390/ijms231710147 (PMC9456285; doi:10.3390/ijms231710147)
Supplement: Supplementary file 1 [file ijms-23-10147-s001.zip › Table S3.pdf]

Table S3 The function of the SR protein in Arabidopsis.

Subfamily	Accession	Nomenclature	Function	Refrences
(1) SR	AT1G09140	AtSR30	light-dependent AS variants	[72, 85]
	AT1G02840	AtSR34	Involvement in ABA-mediated stress responses	[68]
	AT3G49430	AtSR34a		
	AT4G02430	AtSR34b	Involvement in cadmium tolerance, Response to ABA	[68,89]
(2) RSZ	AT1G23860	AtRSZ21	High-temperature responses	[86]
	AT4G31580	AtRSZ22	Interaction with CDKG1 regulating pollen wall formation	[79]
	AT2G24590	AtRSZ22a		
(3) SC	AT5G64200	AtSC35		
(4) SCL	AT5G18810	AtSCL28	Involvement in ABA-mediated stress responses	[68]
	AT3G55460	AtSCL30	Response to environmental stress	[71]
	AT3G13570	AtSCL30a	Involvement in ABA-mediated stress responses	[68]
	AT1G55310	AtSCL33	Involvement in ABA-mediated stress responses	[68]
(5) RS2Z	AT3G53500	AtRS2Z32	Pollen germination and tube growth	[73]
	AT2G37340	AtRS2Z33	Pollen wall formation	[79]
(6) RS	AT2G46610	AtRS31a	Responding to DNA damage from radiation and other stressors inspaceflight	[82]
	AT3G61860	AtRS31	Response to light and sucrose signaling, heat stress	[83, 84]
	AT4G25500	AtRS40	Involvement in ABA-mediated stress response	[68]
	AT5G52040	AtRS41		
(7) SR45	AT1G16610	AtSR45	Response to environmental stress, heat stress Involvement in sugar signaling, and SNF1-related protein kinase 1 stability, flowering	[28,52, 71, 77]
